# Global trends of cardiovascular disease burden attributable to high body mass index from 1990 to 2021 and projections to 2035

**DOI:** 10.3389/fcvm.2025.1700540

**Published:** 2026-01-12

**Authors:** Pingping Huang, Yikun Guo, Gaocan Ren, Lijun Guo, Yifei Wang, Yicheng Liu, Zhibo Zhang, Xiaochang Ma

**Affiliations:** 1 China Academy of Chinese Medical Sciences, Xiyuan Hospital, Beijing, China; 2 China Academy of Chinese Medical Sciences, Graduate School, Beijing, China; 3 Department of Pulmonary, Beijing University of Chinese Medicine Shenzhen Hospital (Longgang), Shenzhen, China; 4 National Clinical Research Center for Chinese Medicine Cardiology, Beijing, China; 5 Xiyuan Hospital, Beijing University of Chinese Medicine, Beijing, China; 6 State Key Laboratory of Traditional Chinese Medicine Syndrome, China Academy of Chinese Medical Sciences, Xiyuan Hospital, Beijing, China

**Keywords:** age–period–cohort study, cardiovascular disease, global burden of disease, high body mass index, Socio-Demographic Index

## Abstract

**Background:**

Cardiovascular disease (CVD) remains the leading cause of mortality and disability globally, with high body mass index (HBMI) playing a pivotal role in its worldwide burden. Gaining a clear understanding and forecasting the effect of HBMI on CVD is crucial for developing effective health policies and interventions.

**Methods:**

We used data from the 2021 Global Burden of Disease study to analyze the CVD burden attributable to HBMI. An age–period–cohort (APC) analysis was conducted to investigate trends in CVD-related mortality attributable to HBMI, whereas the Bayesian Age-Period-Cohort (BAPC) model projected the number of deaths and mortality up to 2035.

**Results:**

The study revealed a significant increase in CVD deaths and disability-adjusted life years (DALYs) due to HBMI globally, despite slightly decreased age-standardized rates (ASR) for HBMI-related CVD. The ASR of deaths and DALYs decreased from 1990 to 2021 in the high and high-middle Socio-Demographic Index (SDI) regions while increasing in the lower SDI regions. A pinpoint analysis revealed the most significant decline in HBMI-related CVD mortality from 2003 to 2010. The BAPC model projected an increase in global HBMI-related CVD deaths to 2,369,451 by 2035. The ASR of deaths is projected to increase to 37.53 per 100,000, with an increase for females and a decrease for males.

**Conclusion:**

This study emphasizes global trends in HBMI-related CVD burden and the importance of targeting HBMI as a modifiable risk factor. It provides crucial information for public health strategies aimed at reducing CVD mortality. Further research is warranted, especially with an aging global population.

## Introduction

Cardiovascular disease (CVD) encompasses a range of conditions affecting the heart and vascular system, including coronary artery disease, myocardial infarction, heart failure, and stroke (1). Typical symptoms of CVD vary by type but may include chest pain, shortness of breath, dizziness, and fatigue, which can lead to life-threatening cardiovascular events if not managed early (2). Globally, CVD remains a leading cause of mortality, imposing a significant social and economic burden. In 2022, global mortality from CVD reached 19.8 million, representing an increase from 12.4 million in 1990 (3). By 2050, it is projected that 61% of adults in the United States will have some form of CVD. The burden of cardiovascular disease will be primarily driven by risk factors such as hypertension, diabetes, and obesity. Globally, more than 1 billion people met the criteria for obesity in 2020 (4).The proportion of individuals with obesity is expected to increase from 43.1% in 2020 to 60.6% by 2050. As a result, the healthcare costs associated with CVD are projected to reach $1.8 trillion (5). With the aging global population, lifestyle changes, and increased metabolic risk factors, CVD incidence and mortality have been rising steadily (6).

High body mass index (HBMI) is a significant risk factor for CVD, influencing both the incidence and progression of various cardiovascular conditions (1). In recent decades, advancements in medical conditions have had a profound impact on managing HBMI and its consequences for CVD. Improved access to obesity management programs, and pharmacological interventions have allowed for better control of risk factors associated with HBMI, leading to a decline in CVD incidence in some populations (7, 8). However, these improvements have not been uniformly distributed, and many low- and middle-income countries continue to face rising rates of obesity and related cardiovascular conditions (9). Understanding the implications of HBMI on CVD development is crucial for analyzing trends and developing targeted interventions. However, existing studies examining CVD burden attributable to HBMI face three critical limitations: crowd restrictions, temporal constraints and methodological narrowness. First, existing studies frequently focus on narrow aspects of the problem or specific populations, failing to provide a holistic understanding of the issue (10, 11). Second, several key analyses concluded in 2019 or earlier (12, 13),omitting critical insights into post-pandemic metabolic risk trends. It is noteworthy that accumulating evidence indicates that the COVID-19 pandemic has exacerbated cardiometabolic risk through decreased physical activity, weight gain and adverse lifestyle changes during lockdowns and quarantine procedures (14–16). Such post-pandemic disruptions further underscore the importance of updating HBMI-related CVD estimates through 2021.In addition, prior studies predominantly focused on point estimates (e.g., incidence rates) without decomposing age-period-cohort (APC) effects, thereby obscuring generational risk trajectories. Furthermore, these analyses were restricted to retrospective calculations of existing data, lacking forward-looking projections of future disease burden (12). This gap limits policymakers’ ability to anticipate resource needs and design proactive interventions for high-risk populations. Therefore, a thorough and systematic analysis of the relationship between HBMI and CVD is essential.

The Global Burden of Disease (GBD) study provides an extensive and authoritative dataset for evaluating global disease burdens. This study, based on the latest GBD 2021 estimates, focuses on analyzing the CVD burden attributable to HBMI, including annual deaths, disability-adjusted life years (DALYs) and annual percentage changes. Additionally, the Bayesian age–period–cohort (BAPC) model was utilized to project future disease burdens. We aimed to evaluate and project the temporal trends of HBMI-induced CVD burden, thereby improving our understanding of future patterns.

## Methods

### Overview

This study used data from the GBD 2021, which provided epidemiological estimates for 371 diseases and injuries (17). We obtained the raw data from the Global Health Data Exchange (GHDx) (https://vizhub.healthdata.org/gbd-results/). The GBD study provides a standardized approach for estimating the absolute numbers, age-standardized rates and crude rates per 100,000 people for prevalence, incidence, mortality, years of life lost (YLL), years lived with disability (YLD) and DALYs. All countries and territories were grouped into 21 regions and then categorized into five groups according to the Socio-Demographic Index (SDI). In addition, the concept of super regions has been introduced, with these regions being classified based on factors such as economic, cultural, social, and geographical characteristics. This classification aims to provide a better understanding of the differences in health burden across various regions (Supplementary Table S1).

### Data source, adjustments and modeling techniques

The GBD estimates were generated through systematic integration of multisource data, rigorous adjustment protocols, and advanced statistical modeling. Data were derived from official statistical systems (civil registrations, health surveys, clinical records, surveillance systems), peer-reviewed literature, and complementary sources (censuses, expert consultations). To address heterogeneity across sources, adjustments included: (1) harmonization of disease definitions, (2) redistribution of misclassified causes of death (e.g., garbage code correction), and (3) statistical imputation for missing data. Modeling leveraged ensemble techniques (CODEm for mortality estimation), Bayesian meta-regression (DisMod-MR for non-fatal outcomes), and spatiotemporal smoothing (ST-GPR for sparse data). Burden metrics (YLD = incidence × duration × disability weight; DALY = YLD + YLL) incorporated demographic references and socioeconomic covariates. All outputs underwent quality control, with 95% uncertainty intervals (95% UI) quantified via Monte Carlo simulations, internal consistency validation, and iterative peer-reviewed refinement (17).

### Definition of the disease concept

HBMI in adults (aged ≥ 20 years) was classified as a BMI of ≥25 kg/m² (18). CVD is a general term for a large disease category, mainly including stroke, ischemic heart disease, hypertensive heart disease and atrial fibrillation/atrial flutter (17).

### The socio-demographic index

The Socio-demographic Index (SDI) is a comprehensive metric introduced by the Institute for Health Metrics and Evaluation (IHME) in 2015. It serves as an indicator to evaluate the development levels of countries or regions, focusing on the intricate association between social progress and population health outcomes. In essence, the SDI is calculated as the geometric mean of three indices—each scaled from 0 to 1—representing the total fertility rate for individuals under 25 years, the average years of education for those aged ≥15 years and the lag-distributed income per capita (PC). The resulting values were multiplied by 100, producing a scale from 0 to 100, in the GBD 2021 study after computing the SDI. On this scale, 0 corresponds to the lowest income, the shortest years of education and the highest fertility rate, whereas 100 reflects the highest income, the longest years of education and the lowest fertility rate. According to their SDI values, 204 countries and territories in GBD 2021 were categorized into five SDI levels: low, low-middle, middle, high-middle and high.

### Disability-adjusted life years

DALYs are a key measure utilized in GBD studies to quantify the overall burden of diseases, injuries and risk factors. It combines the YLL caused by premature mortality and the YLD due to nonfatal health conditions. YLL represents the YLL because of early death compared to the standard life expectancy, whereas YLD accounts for the YLD or ill health. DALYs provide a comprehensive metric to evaluate the health effect of diseases and helps determine priorities for public health interventions.

### Age-standardized rate

Age-Standardized Rate (ASR) is an epidemiological metric that represents the disease burden per 100,000 population, calculated by adjusting crude rates to a standard population structure. This eliminates confounding effects from age distribution differences, enabling unbiased comparisons across populations or time periods.

### Age-period-cohort model

The age-period-cohort (APC) model decomposes temporal trends in population health outcomes into three distinct dimensions: age effects (biological susceptibility variations across lifespan), period effects (external influences uniformly impacting all age groups within specific calendar years, e.g., healthcare reforms or pandemics), and cohort effects (health outcome disparities attributable to shared early-life exposures among birth cohorts). In our GBD analysis, this framework was operationalized through a log-linear regression model:

$$\log(\mathrm{Yi})=\mu +\alpha *\mathrm{ag}e_{i}+\beta *\mathrm{perio}d_{i}+\gamma *\mathrm{cohor}t_{i}+\varepsilon$$

where *Yi* denotes age-specific CVD DALYs or mortality rates, α, β and γ represent the estimable coefficients for age, period, and cohort effects respectively, μ is the intercept, and ε captures residual variance. To address the inherent collinearity between these temporal dimensions (age = period—cohort), we applied the intrinsic estimator (IE) method, which generates unbiased net effects for each dimension through constrained matrix decomposition, aligning with GBD's analytical standards for disentangling complex epidemiological drivers (19).

### Estimated annual percentage change

The estimated annual percentage change (EAPC) is a statistical measure utilized to evaluate the time trend of a specific health indicator, such as incidence, mortality or DALYs, over a given period. EAPC is calculated by fitting a regression model to estimate the average annual rate of change. A positive EAPC indicates an increasing trend, whereas a negative EAPC indicates a decreasing trend over time. This metric is crucial for determining long-term trends in disease burden and assessing the effectiveness of public health interventions.

### Statistical analyses

The assessment of CBD-related disease burden incorporates both incidence rates and absolute case counts of DALYs and mortality. Epidemiologic metrics are standardized as population-weighted estimates per 100,000 individuals for comparative rate analysis, while cumulative case numbers quantify the absolute disease impact. Statistical evaluations were performed through validated analytic approaches with predefined significance thresholds (*α*=0.05). Subsequent sections elaborate on the implementation of specialized modeling techniques, including Bayesian age-period-cohort modeling. Uncertainty Intervals (UIs) represent the range within which the true value of an estimate is expected to fall, typically with a 95% confidence interval (CIs). In GBD studies, CIs are derived using Bayesian hierarchical models, such as those based on Markov Chain Monte Carlo methods to estimate posterior quantiles, nonparametric bootstrap resampling to generate empirical distribution percentiles, or Monte Carlo simulations incorporating stochastic parameter sampling. Key assumptions include appropriate model specifications—for example, the use of Poisson distributions for count data—representative sampling frameworks, and valid distributional approximations. Key tools include DisMod-MR 2.1 (a meta-regression tool with custom MCMC samplers implemented in Python) and spatiotemporal Gaussian process regression (ST-GPR) for covariate adjustment. GBD-reported 95% CIs are interpreted through frequentist frameworks, reflecting repeated-sampling coverage probabilities, or Bayesian frameworks, representing posterior probability intervals. Narrow CIs indicate high precision, whereas wider intervals may reflect data sparsity or heterogeneous uncertainty sources, including measurement error or model misspecification. UIs are generated through a probabilistic Bayesian statistical framework designed to comprehensively quantify uncertainties arising from data inputs, model specifications, and covariate predictions. Unlike CIs, which adopt a frequentist repeated-sampling interpretation and solely reflect sampling variability, GBD UIs provide a Bayesian probabilistic interpretation (a 95% probability that the true value lies within the interval) and systematically account for uncertainties spanning data quality, model structure, and parameter estimation. For this study, we rigorously adhered to the GBD 2021 protocol (17), employing ensemble modeling to harmonize heterogeneous data sources (e.g., vital registration systems, epidemiological surveys).This approach ensures that UIs holistically characterize the layered uncertainties inherent in global burden-of-disease estimates, thereby enhancing their policy relevance and scientific rigor. Data processing and graphical representations were executed utilizing the WHO Health Equity Assessment Toolkit alongside R programming environment (v4.3.2), ensuring methodological consistency across all computational procedures. All statistical data visualizations were performed using JD_GBDR (V2.37, Jingding Medical Technology).

### Bayesian age-period-cohort analysis

We employed the Bayesian age–period–cohort (BAPC) model to project future trends in CVD burden attributable to HBMI. All analyses were conducted in R (version 4.2.3) using the BAPC package together with the R-INLA library for Bayesian inference. This model is well-suited for analyzing complex, high-dimensional data from large-scale epidemiological studies like the GBD 2021, which contains sparse observations across age groups, calendar periods, and birth cohorts. The BAPC model extends the traditional generalized linear model (GLM) within a Bayesian framework, dynamically integrating age, period, and cohort effects using a second-order random walk smoothing approach. Age, period and cohort effects were each assigned second-order random walk (RW2) priors, and the corresponding precision parameters were given weakly informative log-Gamma hyperpriors, which penalize excessive curvature and encourage gradual temporal changes in the latent effects. This allows for continuous temporal evolution of these effects, capturing nonlinear trends and interactions. We approximated the marginal posterior distributions using the Integrated Nested Laplace Approximation (INLA) method, which circumvents computational challenges (e.g., mixing and convergence issues) associated with Markov chain Monte Carlo (MCMC) techniques, ensuring efficient and robust parameter estimation. Leveraging the BAPC R package, we analyzed GBD 2021 data on CVD burden attributable to HBMI, combined with demographic projections from the IHME until 2035. By explicitly modeling the interplay between age, period, and cohort effects, our BAPC framework provides nuanced insights into the evolving global burden of BMI-attributable CVD, informing targeted public health strategies.

## Results

### Global trends in the CVD burden attributable to HBMI

The number of CVD deaths and DALYs attributable to HBMI more than doubled globally from 1990 to 2021. Over the same period, the corresponding age-standardized rates (ASRs) showed only a slight decline. The ASR of HBMI-related CVD deaths decreased from 24.43 per 100,000 in 1990 to 22.77 per 100,000 in 2021, and the ASR of DALYs decreased from 535.01 to 529 per 100,000.

When stratified by sex, both men and women experienced substantial increases in the absolute number of HBMI-related CVD deaths and DALYs. Among men, deaths increased from 389,736 in 1990 to 912,341 in 2021, and DALYs increased from 10,584,855 to 23,957,298. Among women, deaths rose from 473,329 to 991,897, and DALYs rose from 10,497,801 to 21,469,580. Despite these increases in absolute numbers, the ASR of deaths remained relatively stable in both sexes, with men showing higher mortality than women.

Regional patterns were heterogeneous. South Asia showed the largest increases in EAPC for both the ASR of deaths and the ASR of DALYs attributable to HBMI, whereas Australasia exhibited the steepest declines. Detailed estimates by sex, SDI quintile and region are presented in Tables 1, 2.

**Table 1:** The number of Deaths and age standardized Death rate of CVD attributable to HBMI in 1990 and 2021 and its temporal trend from 1990 to 2021, by global, sex, GBD region, and SDI quintile.

Characteristic	1990		2021		1990–2021
	Number of deaths NO. (95% UI)	ASR per 100 000 No. (95% UI)	Number of deaths NO. (95% UI)	ASR per 100 000 No. (95% UI)	EAPC No. (95% CI)
Global	863,065 (477,898,1,316,550)	24.43 (13.63,37.30)	1,904,238 (1,072,732,2,864,241)	22.77 (12.87,34.24)	−0.35 (−0.41to−0.30)
Sex					
Male	389,736 (208,189,598,500)	24.09 (13.11,37.00)	912,341 (478,972,1,393,234)	24.09 (13.20,36.57)	−0.08 (−0.13 to −0.04)
Female	473,329 (268,693,724,371)	24.01 (13.45,36.84)	991,897 (566,163,1,486,812)	21.29 (12.11,31.89)	−0.54 (−0.61 to −0.48)
SDI quintile					
High	277,601 (132,419,453,026)	25.37 (12.02,41.30)	358,201 (198,067,548,404)	16.03 (9.08,24.47)	−1.62 (−1.74 to −1.50)
High middle	288,824 (140,671,460,612)	32.95 (15.91,52.22)	546,184 (277,704,863,727)	28.29 (14.37,44.72)	−0.77 (−1.00 to −0.54)
Middle	167,935 (113,575,237,052)	19.00 (12.81,26.80)	567,898 (322,611,845,036)	22.87 (12.91,34.13)	0.61 (0.52–0.69)
Low middle	94,725 (60,102,134,041)	17.33 (11.15,24.52)	333,449 (193,977,489,319)	24.60 (14.77,35.86)	1.30 (1.25–1.34)
Low	32,179 (21,085,43,123)	15.60 (10.36,20.99)	95,845 (62,834,134,646)	20.61 (13.64,29.36)	0.83 (0.74–0.92)
Regions					
Southeast Asia	25,130 (17,157,34,614)	10.03 (6.83,13.46)	103,564 (63,847,152,176)	16.22 (9.80,23.55)	1.58 (1.48–1.68)
Oceania	1,025 (538,1,677)	34.07 (18.51,55.35)	2,902 (1,411,4,753)	35.30 (18.59,55.86)	0.19 (0.15–0.23)
East Asia	90,898 (64,316,126,194)	13.62 (8.39,19.68)	364,659 (194,222,581,291)	16.37 (8.46,26.45)	1.20 (1.00–1.39)
Eastern Europe	135,165 (47,180,229,422)	52.57 (18.42,90.51)	196,338 (78,766,327,894)	47.49 (18.90,78.77)	−0.33 (−0.88 to −0.22)
Central Asia	23,151 (10,106,37,006)	53.14 (23.53,84.75)	40,400 (19,535,63,842)	52.69 (25.95,84.66)	−0.17 (−0.56 to −0.22)
Central Europe	81,722 (40,098,131,704)	58.62 (28.74,94.65)	100,600 (57,794,154,547)	36.38 (20.57,55.26)	−1.13 (−1.22 to −1.04)
High-income Asia Pacific	11,727 (6,900,17,587)	6.60 (3.80,9.97)	19,560 (10,436,29,956)	2.58 (1.38,3.99)	−1.98 (−2.31 to −1.66)
Australasia	5,734 (2,356,9,628)	25.08 (10.39,42.26)	6,779 (3,166,11,293)	8.67 (4.17,14.36)	−2.71 (−2.83 to −2.60)
Western Europe	140,805 (66,217,227,542)	24.08 (11.30,38.75)	144,491 (78,117,225,161)	10.32 (5.63,15.89)	−2.11 (−2.18 to −2.05)
High-income North America	106,552 (50,480,174,891)	30.24 (14.40,49.34)	161,236 (91,773,242,298)	18.07 (10.40,26.82)	−0.99 (−1.14 to −0.84)
Southern Latin America	13,860 (7,191,22,095)	31.66 (16.48,50.44)	18,341 (10,427,27,595)	17.06 (9.62,25.40)	−1.09 (−1.20 to −0.99)
Caribbean	6,225 (3,488,9,089)	25.16 (14.20,36.75)	15,010 (8,901,22,646)	25.99 (15.32,38.80)	0.43 (0.26 to −0.60)
Andean Latin America	3,256 (1,793,5,062)	16.43 (9.50,25.05)	8,650 (4,687,13,655)	13.86 (7.76,21.58)	−0.26 (−0.46 to −0.06)
Central Latin America	18,922 (10,899,28,642)	24.99 (14.88,38.00)	60,771 (31,551,94,872)	21.87 (11.61,33.87)	−0.21 (−0.44 to −0.02)
Tropical Latin America	25,994 (14,304,39,940)	30.01 (17.21,45.94)	54,963 (31,080,84,783)	18.58 (10.56,28.15)	−1.01 (−1.06 to −0.95)
North Africa and Middle East	94,739 (55,886,140,513)	63.79 (37.96,94.05)	272,085 (153,283,400,687)	68.96 (39.99,101.38)	0.20 (0.11–0.29)
South Asia	25,130 (17,157,34,614)	7.04 (4.25,10.11)	103,564 (63,847,152,176)	15.69 (9.27,23.49)	2.64 (2.54–2.74)
Central Sub-Saharan Africa	46,190 (2,923,6,581)	23.91 (15.30,34.01)	18,185 (10,862,26,012)	47.19 (27.90,70.99)	1.61 (1.57–1.65)
Eastern Sub-Saharan Africa	10,904 7,205,14,505)	16.63 (10.66,23.38)	30,690 (21,259,42,924)	26.22 (16.39,37.42)	0.59 (0.51–0.66)
Southern Sub-Saharan Africa	7,802 (5,361,10,726)	31.25 (21.20,43.42)	25,240 (16,766,35,036)	54.62 (35.04,76.35)	1.68 (1.21–2.14)
Western Sub-Saharan Africa	16,121 (10,935,22,041)	20.30 (13.38,27.77)	48,896 (30,108,71,035)	31.97(19.54,47.20)	0.90 (0.76–1.04)

**Table 2:** The number of DALYs and age standardized DALYs rate of CVD attributable to HBMI in 1990 and 2021 and its temporal trend from 1990 to 2021, by global, sex, GBD region, and SDI quintile.

Characteristic	1990		2021		1990–2021
	Number of DALYs NO. (95% UI)	ASR per 100 000 No. (95% UI)	Number of DALYs NO. (95% UI)	ASR per 100 000 No. (95% UI)	EAPC No. (95% CI)
Global	21,082,656 (11,331,043,32,461,612)	535.01 (291.22,821.15)	45,426,878. (23,767,186,69,603,287)	529.00 (277.28,808.64)	−0.19 (−0.25 to −0.13)
Sex					
Male	10,584,855 (5,472,051,16,280,594)	561.58 (296.68,865.26)	23,957,298 (12,186,757,37,406,115)	587.75 (300.17,913.39)	0.04 (−0.02–0.09)
Female	10,497,801 (5,847,897,16,145,528)	500.11 (279.25,768.29)	21,469,580 (11,681,557,32,032,441)	469.48 (254.30,699.36)	−0.39 (−0.46 to −0.32)
SDI quintile					
High	6,061,548 (2,832,141,9,729,481)	561.14 (262.29,901.02)	7,481,117 (4,022,043,11,418,018)	392.18 (208.92,599.02)	−1.25 (−1.37 to −1.13)
High -middle	6,767,961 (3,125,856,10,909,564)	696.53 (327.00,1,119.01)	11,532,840 (5,403,645,18,590,758)	595.53 (279.91,958.54)	−0.89 (−1.16 to −0.62)
Middle	4,598,335 (2,946,816,6,564,171)	431.50 (287.23,613.19)	14,315,228 (7,452,578,21,827,666)	530.25 (284.53,798.72)	0.65 (0.59–0.71)
Low-middle	2,678,786 (1,658,780,3,853,838)	413.21 (261.10,588.14)	9,244,236 (5,209,810,13,581,708)	608.37 (347.39,888.55)	1.40 (1.35–1.45)
Low	932,976 (616,288,1,270,322)	384.23 (254.52,514.32)	2,795,043 (1,790,707,3,953,351)	501.03 (325.37,702.90)	0.75 (0.67–0.84)
Regions					
Southeast Asia	785,105 (518,959,1,117,790)	271.70 (183.46,378.90)	3,114,699 (1,795,106,4,638,455)	437.64 (257.84,646.45)	1.61 (1.50–1.73)
Oceania	34,888 (17,470,57,339)	969.43 (503.26,1,589.26)	98,570 (46,038,163,498)	1,053.29 (504.47,1,728.29)	0.24 (0.19–0.30)
East Asia	2,321,958 (1,667,594,3,288,831)	278.89 (195.03,391.76)	8,218,277 (4,055,209,13,358,558)	392.39 (197.58,631.99)	1.30 (1.12–1.48)
Eastern Europe	3,117,490 (1,096,166,5,255,899)	1,144.05 (400.42,1,929.40)	4,140,140 (1,642,521,6,909,224)	1,207.62 (475.73,2,015.27)	−0.44 (−1.04 to −0.16)
Central Asia	590,332 (247,462,954,601)	1,247.88 (524.15,2,015.54)	1,001,893 (461,567,1,613,131)	1,223.31 (573.34,1,950.20)	−0.55 (−0.97 to −0.13)
Central Europe	1,881,701 (885,128,3,055,812)	1,280.50 (602.65,2,077.03)	1,885,399 (1,057,878,2,924,068)	864.56 (483.14,1,346.83)	−1.46 (−1.56 to −1.37)
High-income Asia Pacific	257,813 (147,337,394,686)	132.81 (75.94,202.11)	355,589 (180,457,557,572)	84.69 (40.79,134.90)	−1.45 (−1.66 to −1.23)
Australasia	125,318 (50,455,208,796)	542.70 (218.15,902.86)	129,361 (57,338,212,880)	248.67 (108.65,408.01)	−2.67 (−2.82 to −2.52)
Western Europe	2,835,370 (1,293,889,4,580,657)	504.53 (228.45,814.60)	2,372,743 (1,252,565,3,809,676)	249.09 (133.73,398.31)	−2.35 (−2.42 to −2.27)
High-income North America	2,410,149 (1,120,413,3,872,029)	717.07 (334.54,1,150.76)	3,598,605 (2,016,989,5,332,227)	602.11 (343.10,879.10)	−0.71 (−0.83 to −0.60)
Southern Latin America	329,756 (157,821,529,621)	718.55 (346.92,1,155.18)	382,274 (211,200,580,594)	443.99 (242.31,675.09)	−1.33 (−1.42 to −1.25)
Caribbean	163,530 (87,439,241,894)	618.80 (333.01,911.39)	372,304 (210,619,562,112)	694.19 (391.59,1,047.19)	0.50 (0.33–0.67)
Andean Latin America	91,715 (46,412,147,272)	411.82 (218.10,652.07)	221,813 (110,198,357,139)	364.65 (183.35,586.48)	−0.39 (−0.60 to −0.19)
Central Latin America	498,383 (263,728,772,158)	572.50 (316.14,879.27)	1,474,962 (727,346,2,327,248)	580.29 (288.97,913.79)	−0.19 (−0.44 to −0.05)
Tropical Latin America	742,559 (384,529,1,169,441)	755.66 (404.20,1,177.69)	1,406,607 (728,842,2,181,502)	540.61 (281.65,836.09)	−1.14 (−1.20 to −1.09)
North Africa and Middle East	2,589,132 (1,448,194,3,873,128)	1,464.05 (850.77,2,179.82)	7,129,545 (3,799,060,10,548,494)	1,499.72 (823.18,2,207.62)	0.05 (−0.01–0.10)
South Asia	1,197,400 (710,548,1,755,954)	184.29 (110.85,269.01)	6,096,089 (3,097,737,9,141,485)	385.43 (200.83,575.84)	2.63 (2.54–2.72)
Central Sub-Saharan Africa	129,358 (81,295,183,124)	554.54 (358.83,777.80)	503,241 (298,880,732,971)	892.62 (538.65,1,285.60)	1.44 (1.39–1.48)
Eastern Sub-Saharan Africa	315,749 (209,630,413,922)	395.53 (270.72,528.04)	882,359 (596,594,1,201,610)	480.45 (330.93,668.85)	0.46 (0.37–0.54)
Southern Sub-Saharan Africa	221,780 (146,842,304,340)	763.66 (511.49,1,051.32)	654,860 (418,676,914,457)	1,116.80 (720.36,1,551.80)	1.37 (0.93–1.82)
Western Sub-Saharan Africa	443,161 (298,870,621,753)	481.59 (329.16,669.82)	1,387,539 (851,581,2,036,083)	652.42 (400.59,946.98)	0.84 (0.69–0.98)

### Temporal trends in CVD burden attributable to HBMI across age and sex groups

Across age groups, HBMI-related CVD deaths were concentrated in older adults. In 1990, the 75–79 year age group had the highest number of deaths, and the gap between men and women was greatest in this age band. Before 64 years of age, HBMI-related CVD deaths were more frequent in men than in women.

By 2021, the peak in mortality had shifted to the 70–74-year age group, and in this age range women began to surpass men in the number of HBMI-related CVD deaths. For DALYs, the highest burden was observed in the 60–64-year age group in 1990 and in the 65–69-year age group in 2021.

**Figure 1:**
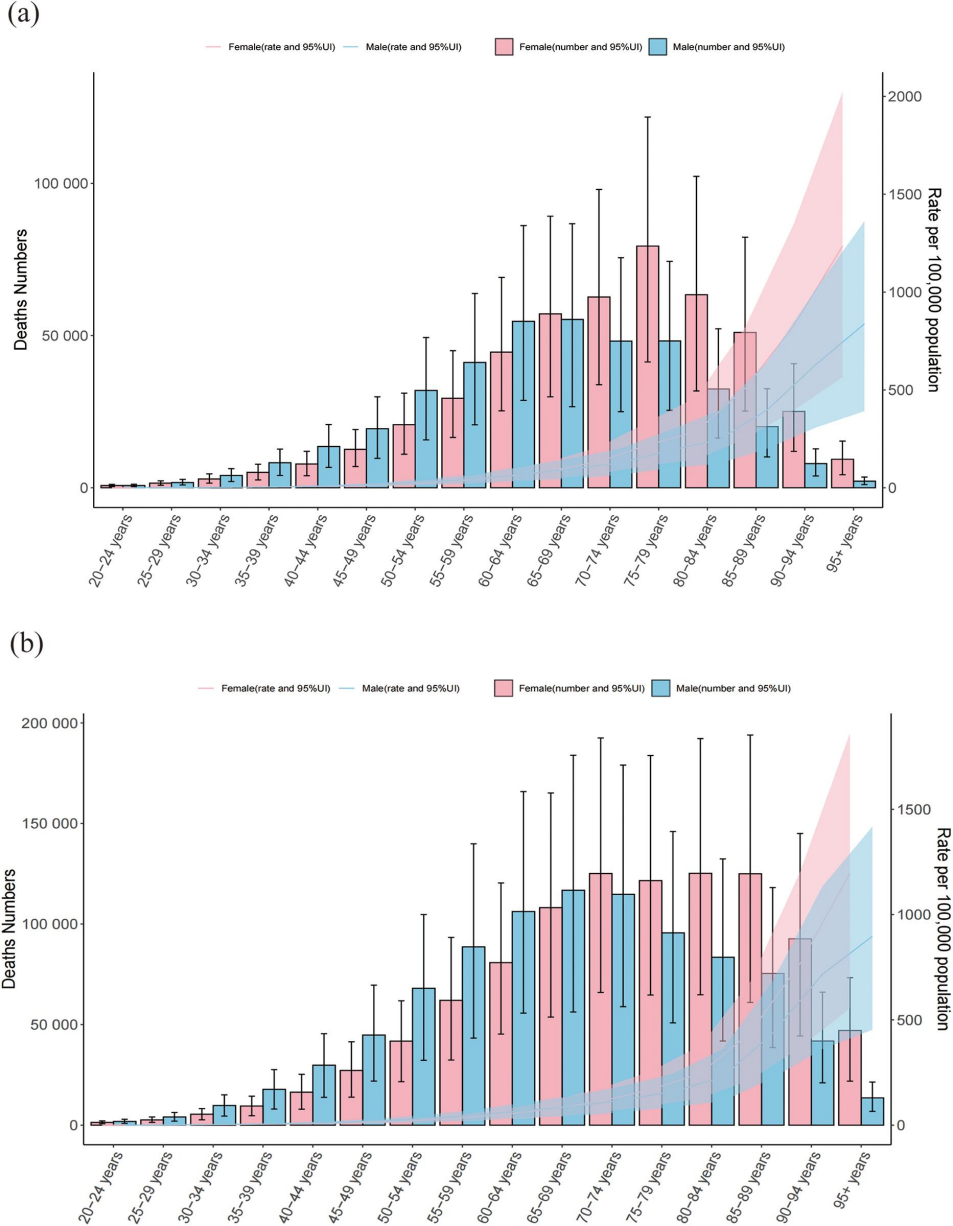
Numbers and ASR of deaths of CVD attributable to HBMI by sex and age in 1990 **(a)** and 2021 **(b)** ASR:age-standardized rate; CVD: cardiovascular disease; HBMI: high body mass index. Shading represents the 95% uncertainty interval (UI) for the rates, while the error bars denote the 95% UI for the numerical values.

**Figure 2:**
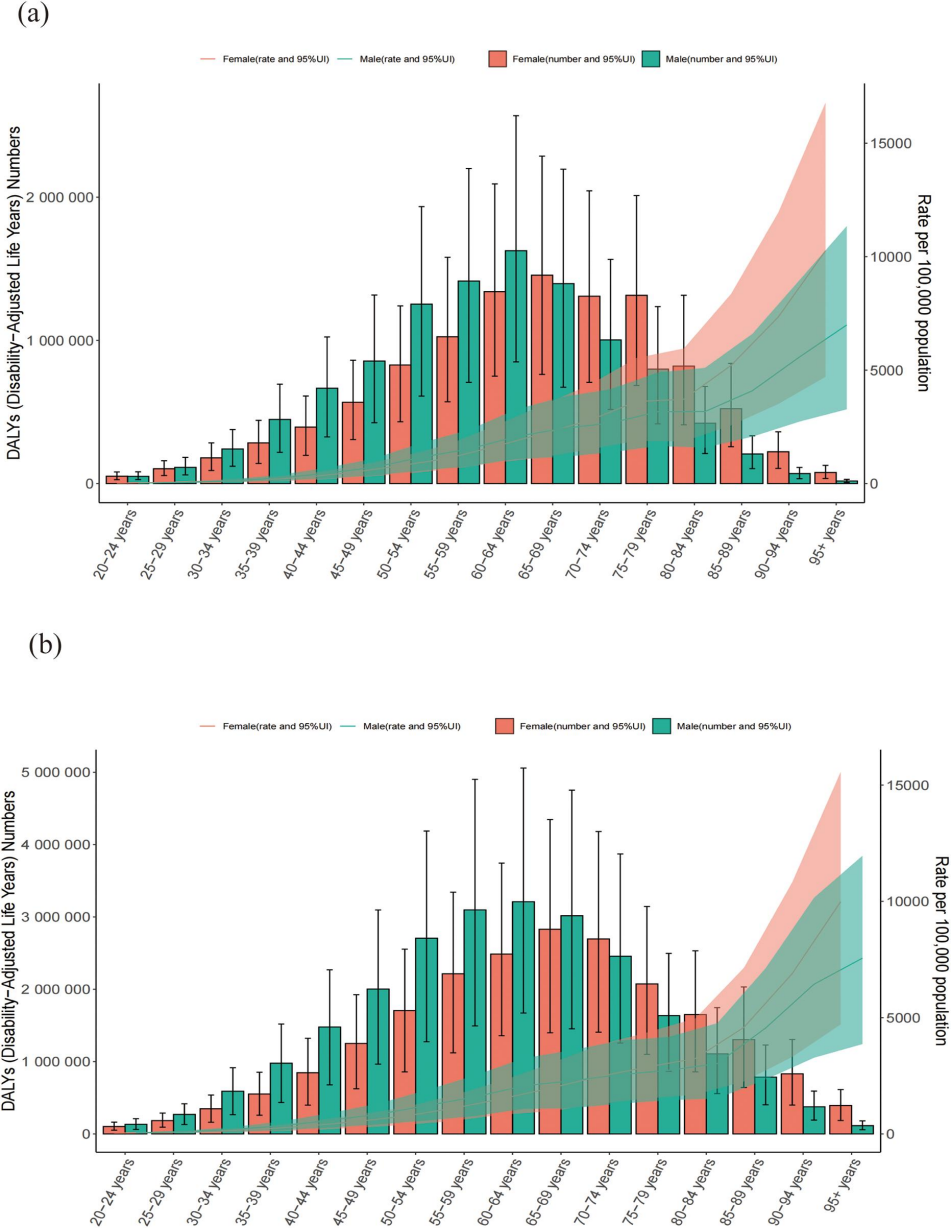
Numbers and ASR of DALYs of CVD attributable to HBMI by sex and age in 1990 **(a)** and 2021 **(b)** ASR: age-standardized rate;CVD: cardiovascular disease; HBMI: high body mass index. Shading represents the 95% uncertainty interval (UI) for the rates, whereas the error bars denote the 95% UI for the numerical values.

Overall, the ASRs of deaths and DALYs increased steadily with age, indicating a stronger effect of HBMI on CVD outcomes in older populations. These age- and sex-specific trends are shown in Figures 1, 2.

### Temporal trends in the CVD burden attributable to HBMI across regional and national groups

**Figure 3:**
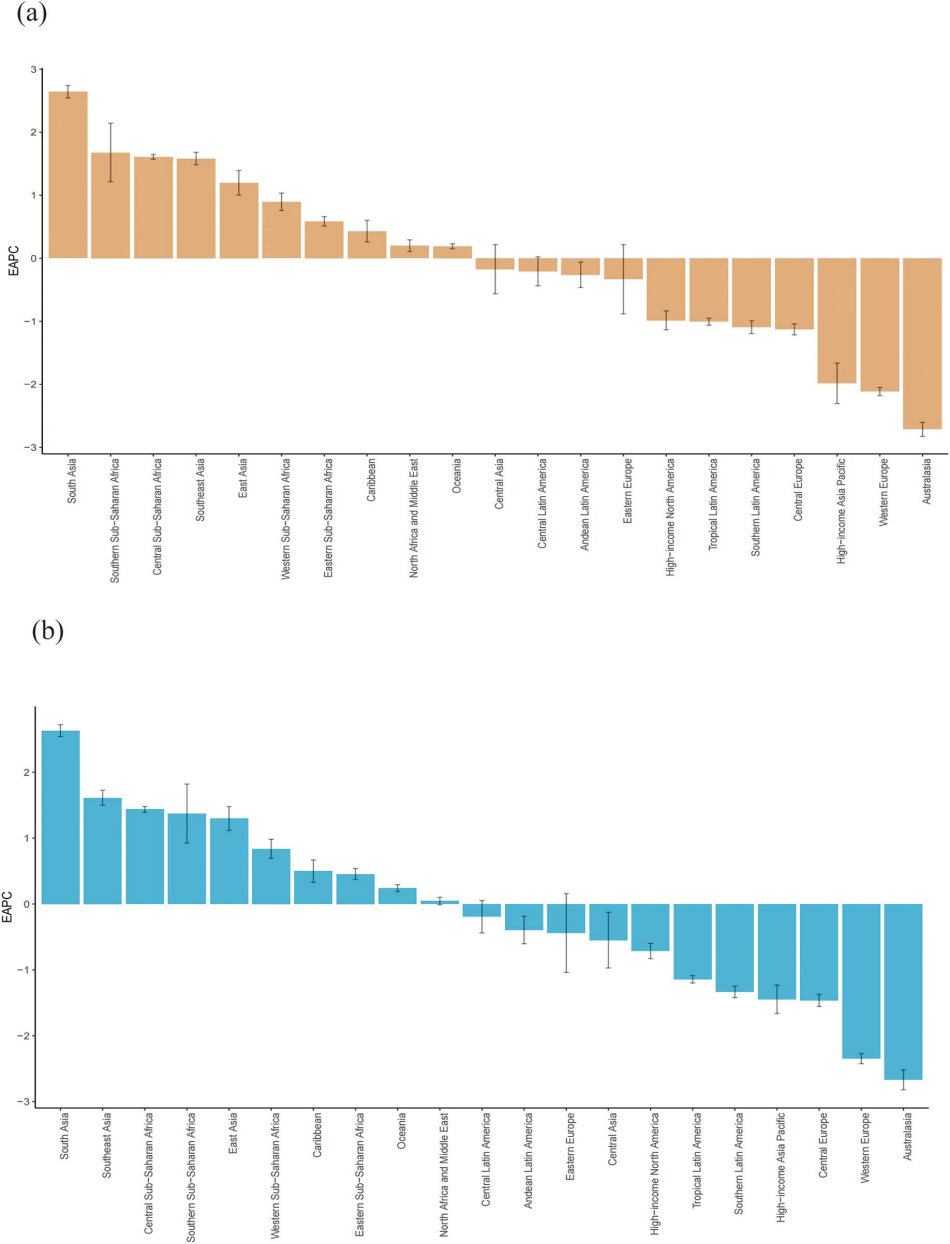
Age-standardized rates of deaths **(a)** and DALYs **(b)** of CVD attributable to HBMI across regional groups. CVD: cardiovascular disease; HBMI: high body mass index. Shading represents the 95% uncertainty interval (UI) for the rates, whereas the error bars denote the 95% UI for the numerical values.

Regionally, from 1990 to 2021, the ASR of deaths due to HBMI-related CVD demonstrated a decreasing trend in 11 out of 21 global regions, whereas 10 regions experienced an increasing trend in their EAPC data. Regarding ASR of DALYs from HBMI-related CVD, all regions demonstrated a decline except for South Asia, Southeast Asia, Central Sub-Saharan Africa, Southern Sub-Saharan Africa, East Asia, Western Sub-Saharan Africa, the Caribbean, Eastern Sub-Saharan Africa, Oceania, North Africa and the Middle East, indicating increasing disease burdens in these regions, which require special attention. Figure 3 presents more detailed information.

Noteworthily, the countries with ASR of CVD deaths attributable to HBMI in 2021 at the national level included the Republic of Nauru, the Arab Republic of Egypt, the Syrian Arab Republic and the Republic of Bulgaria. The leading countries were the Republic of Nauru, the Arab Republic of Egypt, the Federated States of Micronesia, the Republic of the Marshall Islands and Tuvalu in terms of DALYs. The Republic of Zimbabwe demonstrated the largest increase in the EAPC for both ASR of deaths and DALYs, whereas the State of Israel saw the smallest increase.

**Figure 4:**
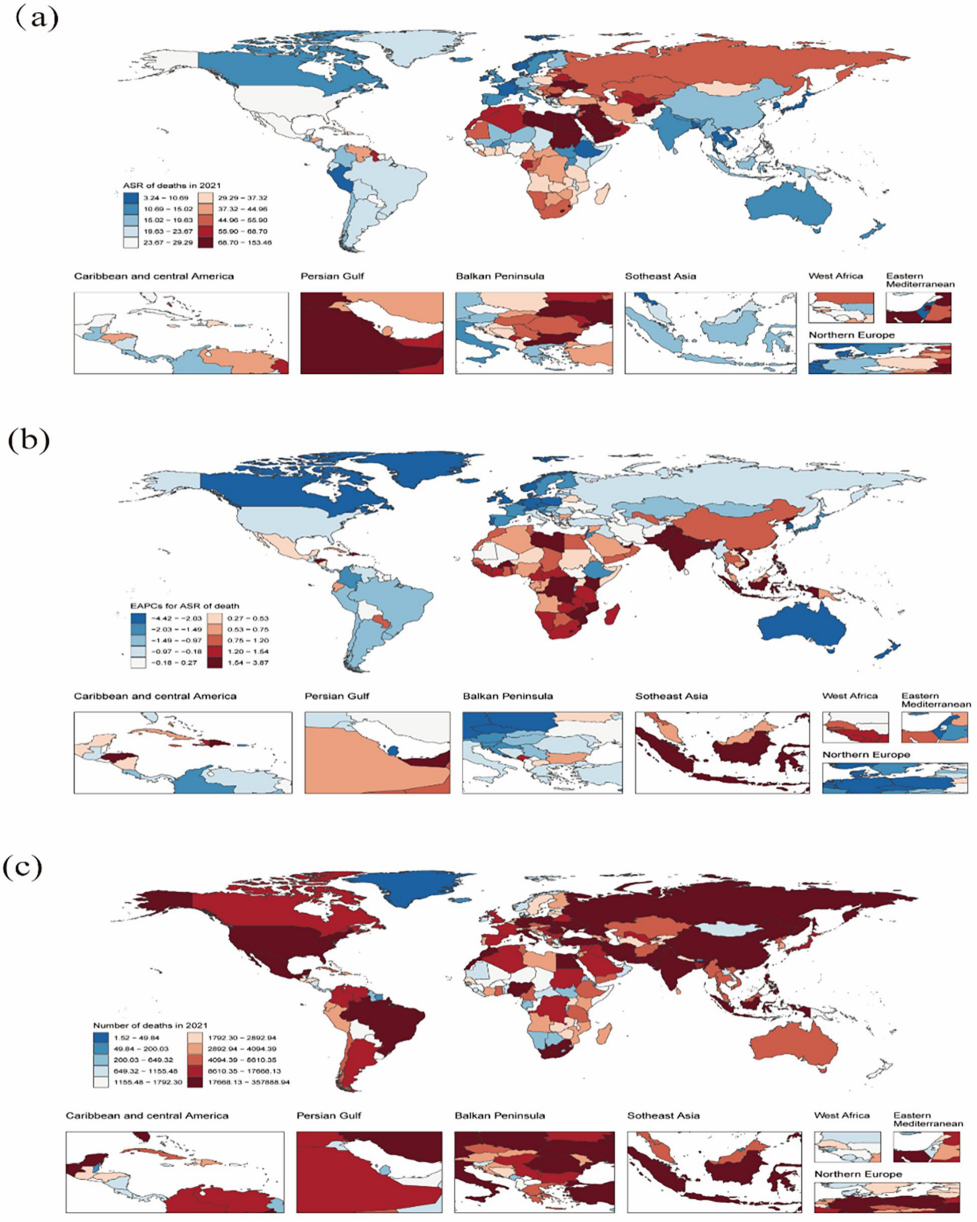
Global death burden of CVD attributable to HBMI in 204 countries and territories. **(a)** ASR of deaths in 2021. **(b)** EAPC in the ASR of deaths of CVD attributable to HBMI from 1990 to 2021. **(c)** The absolute number of deaths in 2021. ASR: age-standardized rate; CVD: cardiovascular disease; HBMI: high body mass index; EAPC: estimated annual percentage change. Map generated using the R software package (version 4.2.3) and JD_GBDR (V2.22, Jingding Medical Technology). Map generated using the rnaturalearth R package developed and maintained by Andy South et al. Map data adapted from Natural Earth (https://www.naturalearthdata.com/).

**Figure 5:**
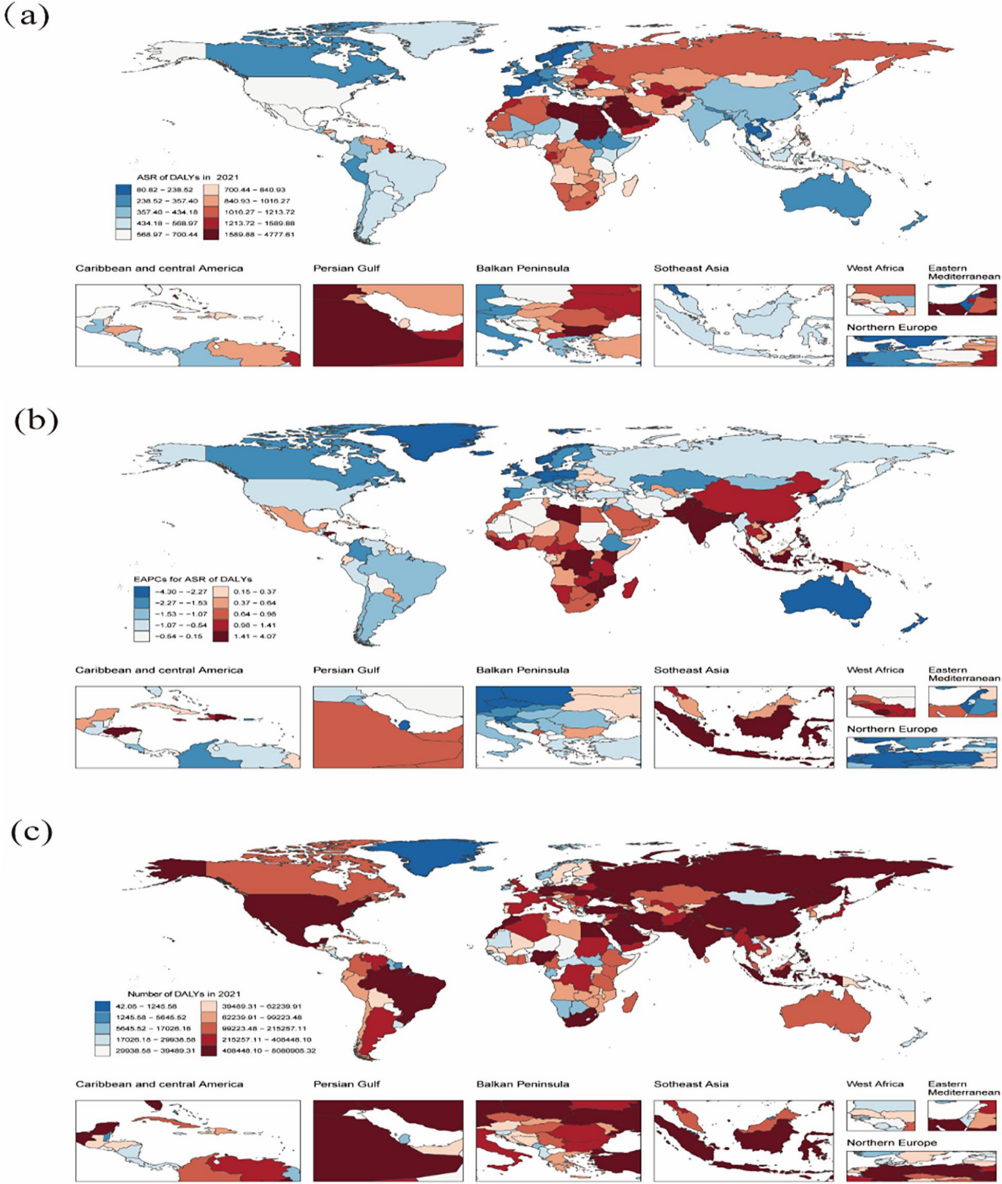
The global DALYs burden of CVD attributable to HBMI in 204 countries and territories. **(a)** ASR of DALYs in 2021. **(b)** EAPC in ASR of DALYs of CVD attributable to HBMI from 1990 to 2021. **(c)** The absolute number of DALYs in 2021. DALYs: disability-adjusted life years; CVD: cardiovascular disease; HBMI: high body mass index; EAPC: estimated annual percentage change. Map generated using the R software package (version4.2.3) and JD_GBDR (V2.22, Jingding Medical Technology). Map generated using the rnaturalearth R package developed and maintained by Andy South et al. Map data adapted from Natural Earth (https://www.naturalearthdata.com/).

Additionally, the countries with the highest growth in EAPC for ASR of deaths and DALYs from 1990 to 2021 included the Republic of Zimbabwe, the Kingdom of Lesotho, the Republic of Indonesia and the Republic of India. In 2021, the countries with the highest number of CVD deaths and DALYs due to HBMI were China, Russia, India and the United States. Overall, the number of deaths and DALYs appeared to be strongly associated with the population size. Supplementary Tables S2, S3 and Figures 4,5 present more information.

### Trends in the CVD burden attributable to HBMI across socio-demographic index quintiles

**Figure 6:**
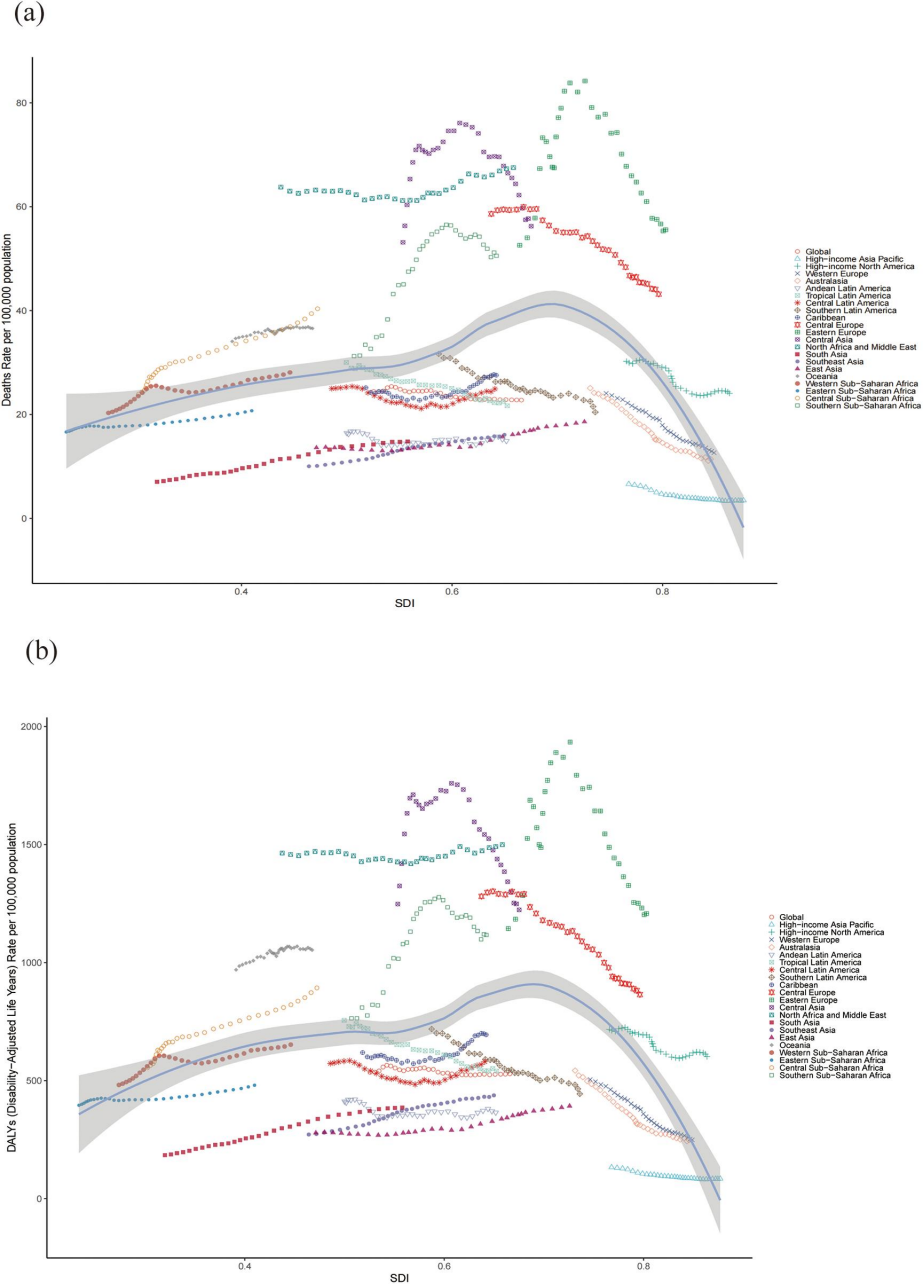
ASR of CVD-related deaths **(a)** and DALYs **(b)** attributable to HBMI across 21 regions stratified by SDI. The points plotted from left to right represent the annual estimates from 1990 to 2021 for each region. CVD: cardiovascular disease; ASR: age-standardized rate; DALYs: disability-adjusted life years; SDI: Socio-demographic Index.

Significant changes in the ASR for CVD attributable to HBMI were observed globally over the 31 years of the study. The results revealed a consistent trend when classified by SDI levels: the EAPC for ASR of death and DALYs decreased from 1990 to 2021 in the high and high-middle SDI regions, whereas these indicators increased in middle, low-middle and low SDI regions. Across all regions, the ASR for HBMI-related CVD deaths and DALYs increased with increasing SDI, up to an approximate SDI threshold of >0.70, after which it decreased. Tables 1, 2 and Figure 6 show the results.

Notably, among the four regions with the highest SDI scores in 2021, three demonstrated a continuous decline in the ASR of death and DALYs from HBMI-related cardiovascular disease over the past 31 years, whereas high-income North America exhibited a fluctuating downward trend. The EAPC of the ASR of death and DALYs in these regions are remarkable. For death: high-income Asia Pacific [−1.98 (95% CI: −2.31 to −1.66)], Australasia [−2.71 (95% CI: −2.83 to −2.60)], high-income North America [−0.99 (95% CI: −1.14 to −0.84)] and Western Europe [−2.11 (95% CI: −2.18 to −2.05)]. For DALYs: high-income Asia Pacific [−1.45 (95% CI: −1.66 to −1.23)], Australasia [−2.67 (95% CI: −2.82 to −2.52)], high-income North America [−0.71 (95% CI: −0.83 to −0.60)] and Western Europe [−2.35 (95% CI: −2.42 to −2.27)]. Tables 1, 2 and Figure 6 present the results.

### Age–period–cohort effects on CVD burden attributable to HBMI

Net drift represents the overall annual percentage change, where a value of <0 indicates a significant reduction in CVD mortality over the study period. Conversely, local drift values represent the annual percentage change for each age group. Both local drift values of <0 indicate a downward trend in CVD mortality across all age groups throughout the study period.

In general, the net drift of CVD mortality [−0.16% (95% CI: −0.23% to −0.1%) per year] is attributable to HBMI in the global region based on the age–period–cohort model.

The longitudinal age distribution of CVD-related mortality indicates an increase in mortality rates across all age groups in individuals with a high body mass index. The pattern of increasing mortality was similar for both males and females, with a particularly sharp rise in mortality risk beginning from the 60 age group.

**Figure 7:**
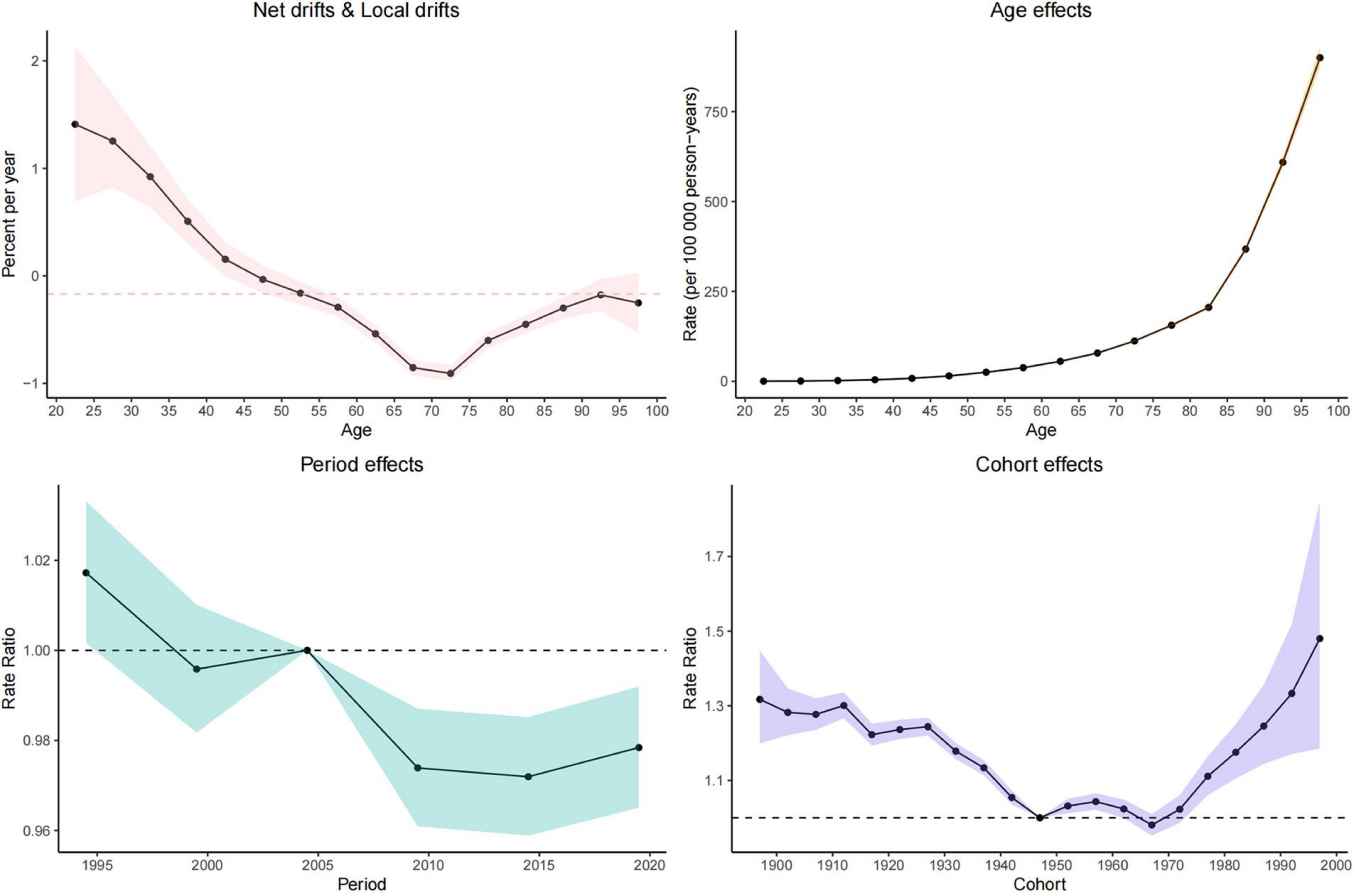
APC model of CVD-related mortality attributable to HBMI.

Regarding the period effect, the trend of CVD-related mortality risk in the overall population with HBMI first showed a decreasing trend and then gradually stabilized. The cohort effect indicates that the younger cohorts born after 1980 show an increasing risk of CVD attributable to HBMI. Figure 7 shows detailed information.

### The average annual percentage change model effects on the CVD burden attributable to HBMI

**Figure 8:**
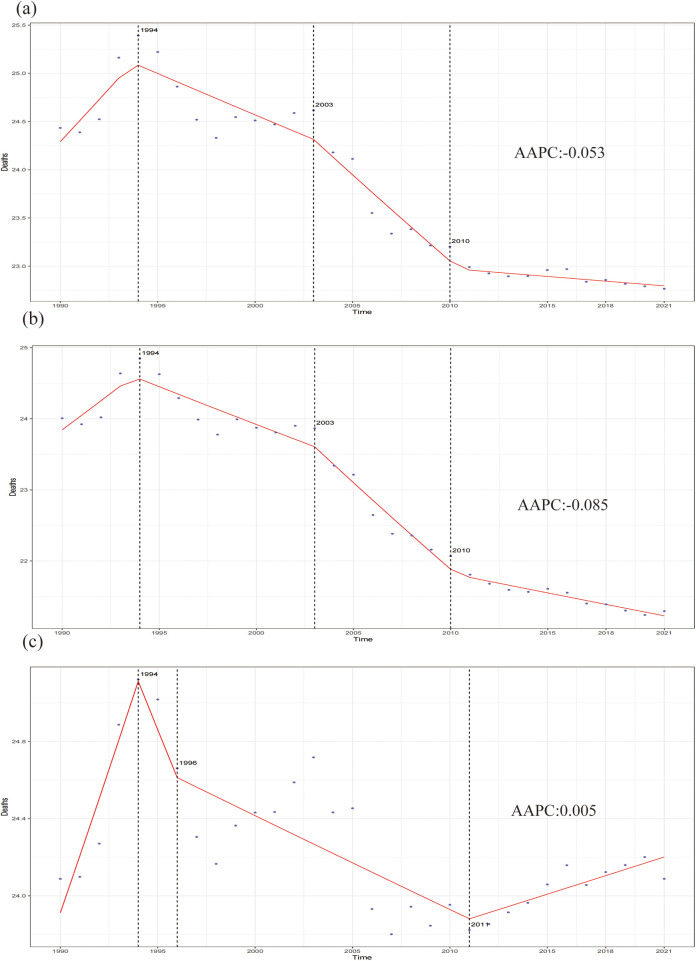
The pinpoint regression analysis of the age-standardized death rate for CVD attributable to HBMI from 1990 to 2021. **(a)** Total population. **(b)** Female. **(c)** Male.

Figure 8 illustrates the 31-year trend in the ASR of deaths attributable to HBMI in the overall population. The global ASR of deaths attributable to HBMI from 1990 to 2021 can be divided into four phases, with the third phase demonstrating a notable decline. Specifically, from 2003 to 2010, the mortality rate decreased by 0.75%, with an overall AAPC of −0.053 for the entire period. A similar trend was observed in females, with a significant decline of 1.08% from 2003 to 2010 and an AAPC of −0.085. In contrast, males demonstrated a fluctuating upward trend, with a notable decrease of −0.20% in the third phase and an AAPC of 0.005 over the period. Supplementary Table S4 presents further details.

### Projected trends in the CVD burden attributable to HBMI: insights from the BAPC model

**Figure 9:**
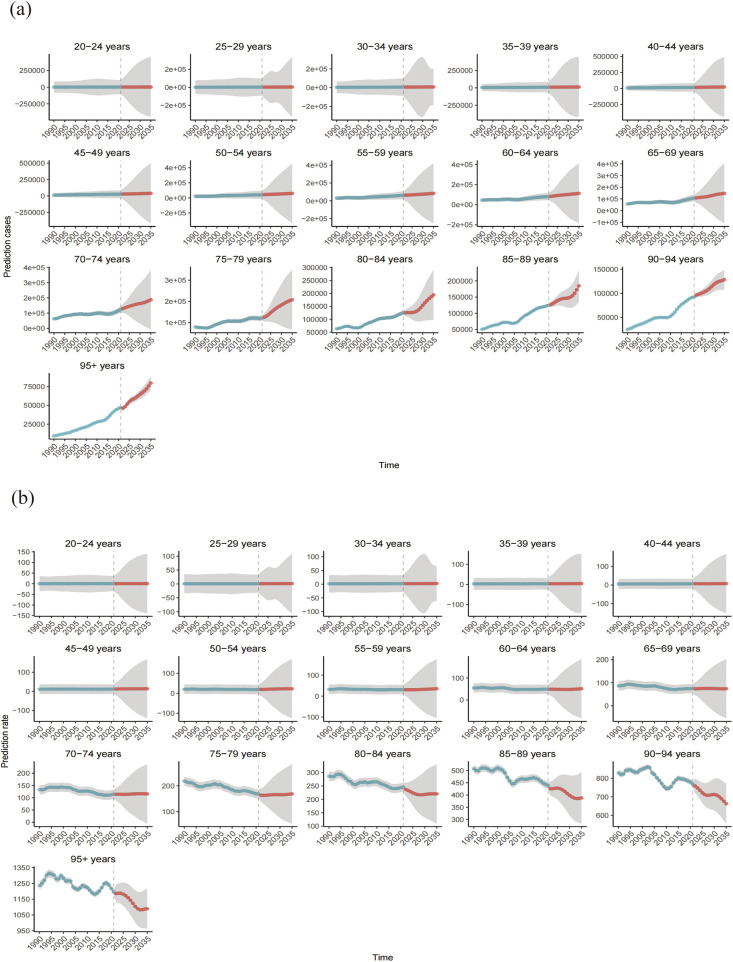
Number of deaths **(a)** and mortality rate **(b)** of CVD attributable to HBMI from 1990 to 2035.Shaded grey areas denote the 95% uncertainty intervals (UIs) derived from the posterior distribution.

**Figure 10:**
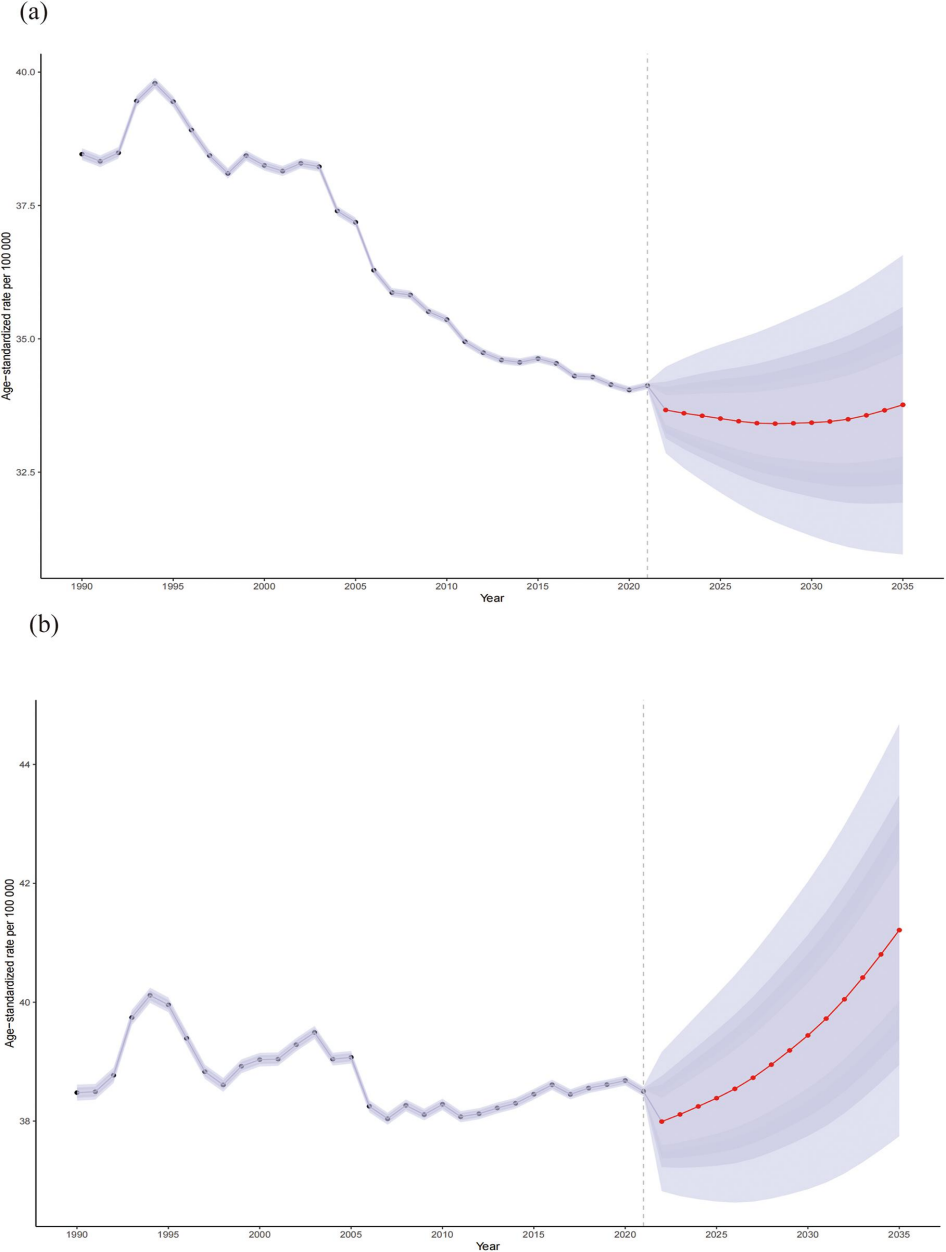
Sex differences (female: **a**; male: **b**) in the mortality rates of CVD attributable to HBMI from 1990 to 2035. Shaded purple areas denote the 95% Uncertainty Intervals (UIs) derived from the posterior distribution.

The number of global CVD deaths attributable to HBMI is projected to increase to 2,369,451 (95% UI: 2,163,223.82 to 2,575,678.63) by 2035, reflecting an increase of over 20% compared to 2021, with the ASR expected to reach 37.53(95% UI: 34.27–40.80) per 100,000. Across age groups, the 20–59-year age group remained relatively stable, whereas deaths increased significantly after 60 years of age. However, ASR trends differed, remaining stable between ages 20 and 79 years but exhibiting a decline followed by stabilization after 80 years of age. Additionally, we predict an increasing ASR trend in males and a declining trend in females. Figures 9, 10 and Supplementary Table S5 show more details.

## Discussion

CVD imposes a significant global health burden, and HBMI is a crucial modifiable risk factor, drawing considerable attention. To the best of our knowledge, this is the first longitudinal analysis using the BAPC model based on GBD 2021 data combined with pinpoint regression analysis to investigate global trends in CVD caused by HBMI. The results emphasize population aging and low SDI areas as key contributors to the CVD burden, with notable variations across countries and regions, providing valuable information for shaping public health policies and interventions.

Population ageing emerged as a central driver of the HBMI-attributable CVD burden in our analysis. Although the numbers of CVD deaths and DALYs attributable to HBMI increased markedly from 1990 to 2021, the corresponding ASRs declined only slightly, and our projections suggest that this divergence between absolute burden and standardized rates will persist until 2035, indicating that the expanding pool of older adults at risk, rather than rising age-specific rates, is the main contributor to the increase in events. This pattern should be interpreted in the context of rapid global demographic change: individuals aged ≥70 and ≥90 years are among the fastest-growing age groups in Europe, Asia and the United States, and the proportion of older adults in the global population is projected to rise from 5% in 1950 to 16% by 2050 (20). In this setting, HBMI-related CVD is expected to become particularly burdensome in middle- and low–middle-SDI regions, where health infrastructure and health promotion remain limited (21). In our study, HBMI-related CVD deaths among individuals aged ≥60 years increased steadily over time (Figure 1), consistent with global ageing trends. For example, in India, people aged ≥60 years accounted for 9.9% of the population (133.32 million) in 2021, a proportion projected to rise to 17.3% (300.96 million) by 2050 (22). The biological plausibility of these findings is supported by evidence that ageing is accompanied by immune dysfunction and chronic low-grade inflammation in adipose tissue, which accelerates vascular ageing in individuals with obesity (23), as well as reduced metabolic capacity and a shift towards central fat accumulation, both of which heighten CVD risk (24). Moreover, HBMI in late life frequently coexists with hypertension (25) and diabetes (26), leading to clustering of metabolic risks. In elderly patients with ischaemic heart disease in Iran, systolic blood pressure, low-density lipoprotein cholesterol and hyperglycaemia together accounted for 820.4 thousand DALYs nationwide (27).

In addition to population aging, this study also revealed significant regional inequality in the distribution of disease burden. the ASR growth in low SDI regions contrasts sharply with the decline in high SDI regions. This trend is driven by a combination of social, economic and behavioral factors. First, rapid urbanization has caused significant lifestyle changes in these areas, with decreased physical activity and increased sedentary behavior, both major contributors to the rise in HBMI and subsequent CVD. Luo et al. emphasized the crucial role of low physical activity in the growing CVD burden in low SDI regions (28). Second, traditional diets are being replaced by processed foods high in sugar and unhealthy fats with economic development and the expansion of the food industry, thereby further promoting obesity and CVD. Zhang revealed the significant effect of poor dietary habits on increasing CVD mortality in low-middle SDI regions (29). Additionally, weaker healthcare infrastructure in these areas has delayed the early diagnosis and treatment of obesity and CVD, exacerbating the disease burden due to a lack of timely interventions. Shi underscored the important role that healthcare system limitations play in increasing the disease burden in these regions (30). China provides a representative example of these patterns. Using GBD 2019 data, Zhang et al. reported that (31), although age-standardized CVD rates in China have started to decline, the absolute numbers of CVD cases, deaths and DALYs continued to increase between 1990 and 2019, particularly among older adults and residents of less developed provinces, largely driven by metabolic and behavioral risks. This is consistent with our finding that China had relatively high absolute numbers of HBMI-attributable CVD deaths and DALYs globally in 2021, highlighting the need to strengthen cardiometabolic prevention in middle-SDI settings.

Beyond age and regional disparities, we also observed pronounced sex-specific patterns in the HBMI-attributable CVD burden. Longitudinal analyses using AAPC, APC and BAPC models showed that, although overall ASRs changed only modestly, HBMI-related CVD mortality is projected to decrease in women but increase in men, suggesting divergent future trajectories. This pattern is biologically and behaviorally plausible. Men and women differ in fat distribution, metabolism and immune responses (32, 33). Men tend to accumulate more visceral abdominal fat, which is strongly associated with CVD, whereas women have a higher proportion of subcutaneous fat, which has a weaker impact on cardiovascular risk and may partly contribute to the decline in female mortality (34, 35). Behavioural profiles also differ. Men are more likely to smoke and consume alcohol excessively (36, 37) They are also less engaged in early preventive care and regular health monitoring and often face greater work- and finance-related stress with relatively limited social support. By contrast, women more frequently seek preventive services and pay closer attention to weight control and healthy eating, and they may benefit from broader social support networks (38, 39). We further observed that men reached peak HBMI-related CVD mortality at younger ages than women, consistent with the cardioprotective effects of oestrogen before menopause and the sharp rise in women's CVD risk after menopause (40). Stroke, a major HBMI-sensitive CVD subtype, shows a similar pattern in China. Based on GBD 2021 data, Ji et al. reported that (41) stroke prevalence more than doubled between 1990 and 2021, with 2.59 million deaths and a 45% increase in DALYs, with increases more pronounced in men. They also found that metabolic risks remained the leading contributors to stroke mortality and the proportion of stroke deaths attributable to high BMI rose from 0.34% to 2.74% over this period. Taken together, these findings support the conclusion that effective control of BMI and other metabolic factors in middle-aged and older adults, particularly men, will be critical for reducing the future HBMI-related CVD burden.

Collectively, the observed and projected patterns of HBMI-related CVD burden suggest that interventions should preferentially target two groups: populations residing in low-SDI regions and adults aged ≥60 years. These groups bear disproportionately heavier impacts, underscoring the urgent need for targeted health interventions in economically disadvantaged areas and aging populations. In low SDI settings, healthcare systems should prioritize early CVD screening for older adults combined with BMI monitoring to implement personalized interventions such as regular health assessments, weight management programs, and nutritional counseling. The rising ASR trends among middle-aged males necessitate enhanced health education initiatives, particularly workplace- and community-based programs promoting physical activity and dietary modifications. While females demonstrate declining ASR trends, persistent cardiovascular risks associated with elevated BMI in older women warrant intensified weight control strategies and lifestyle interventions targeting this subgroup. Furthermore, low SDI regions should implement localized health policies to improve dietary environments through sugar/fat intake reduction campaigns and expand accessible exercise infrastructure, with special provisions for low-income populations to ensure equitable access to health promotion resources. These multifaceted strategies—spanning precision screening, gender-specific prevention, and environmental modifications—could translate epidemiological insights into actionable plans to mitigate HBMI-related CVD morbidity and mortality. By integrating age-stratified interventions with regional socioeconomic considerations, such approaches align with global health equity goals while addressing the dual challenges of population aging and nutritional transitions.

This paper has several strengths. First, it used the GBD2021 dataset, which covers multiple countries and regions, including CVD data from various age groups, periods and cohorts globally. These extensive global data enable the identification of trends across regions and provide valuable information for global and regional health policy recommendations. Second, this study spans 31 years from 1990 to 2021, enabling a long-term analysis of trends in CVD burden and the effect of HBMI, revealing how these factors evolve. Additionally, the APC model distinguishes between age, period and cohort effects, enabling the study to uncover health changes over time within different generations (cohorts). This model not only accounts for age-related risks but also considers the effect of specific periods and environmental factors on population health. Furthermore, we applied the BAPC model to predict future disease burden, providing stakeholders with valuable information to accurately anticipate disease trends. Finally, the research provides empirical data for policymakers by discussing the effect of high BMI on the CVD burden, thereby helping them prioritize BMI management in prevention and intervention strategies, particularly those targeting younger generations and specific periods.

However, this paper has several limitations. To begin with, this analysis relies on aggregated GBD 2021 estimates, which may be influenced by regional variations in data quality. Low-SDI regions frequently experience incomplete vital registration and underreporting of causes of death, which, despite GBD's standardized correction algorithms, may introduce uncertainty into HBMI-related CVD estimates. These factors may particularly affect absolute burden estimates in settings with sparse input data, although overall temporal patterns are likely to remain robust. In particular, some low-income countries may lack sufficient health monitoring systems or high-quality cause-of-death reporting, resulting in data imbalance or bias. Moreover, the definition of HBMI and the standardized methods utilized may vary across regions. Populations in certain areas may demonstrate different body composition baselines, which may cause BMI to have a varied effect on disease risk across regions. Furthermore, the macrolevel GBD data cannot capture individual behavioral dynamics. For example, APC analysis cannot account for individual lifestyle changes (such as exercise, diet or healthcare behavior), which significantly affect BMI and CVD risk. In addition, the study did not capture the interaction between BMI and other health factors. The focus is on the effect of HBMI on CVD, but BMI frequently interacts with other risk factors (such as hypertension, diabetes and smoking), and these complex interactions may not be fully reflected or may be overlooked in the analysis. Another important limitation is that, our projections depend on demographic inputs derived from IHME population forecasts. While these projections are widely used and methodologically rigorous, long-term estimates may still be sensitive to underlying assumptions regarding fertility, mortality, and migration trends. Therefore, the interpretation of disease burden projections beyond 2030 should consider potential uncertainties embedded in demographic priors. Finally, although our analysis provides a comprehensive overview of the HBMI-attributable CVD burden, we were unable to disaggregate trends by specific CVD subtypes such as stroke or ischemic heart disease due to inconsistencies in long-term annual subtype estimates across regions in the GBD 2021 dataset. Subtype-specific APC and BAPC analyses may reveal differential HBMI-related impacts and represent an important direction for future research.

## Conclusions

This study first deconstructed the intergenerational, temporal, and age effects of HBMI related CVD burden from 1990 to 2035 using the APC and BAPC model, revealing that low SDI areas and aging populations are the core targets for future interventions. The results call for the inclusion of BMI control in the global cardiovascular disease prevention and control system, with particular attention to middle-aged and elderly men and the nutritional transition in rapidly urbanized areas.

## Data Availability

The datasets generated and/or analyzed in the present study are available from the corresponding author upon reasonable request.
